# Simple Mobile technology health management tool for people with severe mental illness: a randomised controlled feasibility trial

**DOI:** 10.1186/s12888-021-03359-z

**Published:** 2021-07-16

**Authors:** Frank Röhricht, Raguraman Padmanabhan, Paul Binfield, Deepa Mavji, Sally Barlow

**Affiliations:** 1 East London NHS Foundation Trust, London, UK; 2 Wolfson Institute for Preventive Medicine, Queen Mary University of London, London, UK; 3 Centre for Mental Health Research, School of Health Sciences, City University of London, London, UK

**Keywords:** Mobile health technology, Recovery care, Self-management, Severe mental illness, RCT

## Abstract

**Background:**

Severe mental illness (SMI) is associated with care delivery problems because of the high levels of clinical resources needed to address patient’s psychosocial impairment and to support inclusion in society. Current routine appointment systems do not adequately foster recovery care and are not systematically capturing information suggestive of urgent care needs. This study aimed to assess the feasibility, acceptability, and potential clinical benefits of a mobile technology health management tool to enhance community care for people with severe mental illness.

**Methods:**

This randomised-controlled feasibility pilot study utilised mixed quantitative (measure on subjective quality of life as primary outcome; questionnaires on self-management skills, medication adherence scale as secondary outcomes) and qualitative (thematic analysis) methodologies. The intervention was a simple interactive technology (Short Message Service - SMS) communication system called ‘Florence’, and had three components: medication and appointment reminders, daily individually defined wellbeing scores and optionally coded request for additional support. Eligible participants (diagnosed with schizophrenia, schizoaffective disorder or bipolar disorder ≥1 year) were randomised (1:1) to either treatment as usual (TAU, *N* = 29) or TAU and the technology-assisted intervention (*N* = 36).

**Results:**

Preliminary results suggest that the health technology tool appeared to offer a practicable and acceptable intervention for patients with SMI in managing their condition. Recruitment and retention data indicated feasibility, the qualitative analysis identified suggestions for further improvement of the intervention. Patients engaged well and benefited from SMS reminders and from monitoring their individual wellbeing scores; recommendations were made to further personalise the intervention. The care coordinators did not utilise aspects of the intervention per protocol due to a variety of organisational barriers. Quantitative analysis of outcomes (including a patient-reported outcome measure on subjective quality of life, self-efficacy/competence and medication adherence measures) did not identify significant changes between groups over time in favour of the Florence intervention, given high baseline scores. The wellbeing scores, however, were positively correlated with all outcome measures.

**Conclusion:**

It is feasible to conduct an adequately powered full trial to evaluate this intervention. Inclusion criteria should be revised to include patients with a higher level of need and clinicians should receive more in-depth assistance in managing the tools effectively. The preliminary data suggests that this intervention can aid recovery care and individually defined wellbeing scores are highly predictive of a range of recovery outcomes; they could, therefore, guide the allocation of routine care resources.

**Trial registration:**

ISRCTN34124141; retrospectively registered, date of registration 05/11/2019.

## Background

Between 0.5 and 1% of the population experience severe mental illness (SMI) during their lifetime and about a third of patients develop a more chronic course of their illness, in particular those with chronic psychosis (Schizophrenia, Schizoaffective Disorder, Bipolar Disorder) [1]. A high percentage of these patients continue to have poor outcomes, including social isolation, medical comorbidity, and poor quality of life [2–6].

Due to the complexity of the illness patients often require help and support from a range of health and social care professionals, which may result in problems with the coordination and timely delivery of all the care components. Many studies identified that people living with SMI struggle to comply with their treatment over time, and a significant number of people disengage from services [7–9]. A recent systematic review identified a negative attitude toward medication as a key reason directly associated with intentional non-adherence. Negative attitude to medication was also associated with level of insight and the quality of the therapeutic alliance [10]. Several of these factors are modifiable, and therefore, interventions aiming to enhance patient’s self-management skills, knowledge, and motivation may help improve overall treatment adherence. Levin et al. (2016) emphasised that “for adherence-enhancement approaches to be widely adapted, they need to be easily accessible, affordable, and practical” [11, p. 819]. System support factors such as shared decision making, the impact of side effects, and the way patients are provided with information about medication and their potential side effects have been discussed in the literature as compounding factors for treatment adherence [e.g. 10, 11].

Current routine appointment systems do not sufficiently provide for immediate care needs in a timely fashion and patients have little control over their care arrangements, while the recovery care agenda emphasised the importance of empowerment for people with SMI [12]. In addition, enduring conditions such as SMIs carry a high economic burden with significant costs to the NHS; accounting for approximately £2.4–2.8 billion total annual cost to the English NHS [13]. New and cost-effective ways of delivering integrated health and social care for patients with SMI are required. Lean et al. (2019) conducted a systematic review and meta-analysis of research on self-management for people with severe mental illness and found that “there is evidence that the provision of self-management interventions alongside standard care improves outcomes for people with SMI”; they concluded that these interventions “should form part of the standard package of care provided to people with SMI and should be prioritised in guidelines: research on best methods of implementing such interventions in routine practice is needed” [14 , p. 260].

Mobile health (“m-health”) technology has been increasingly proposed and tested as possible clinical and cost-effective solutions to foster self-management, monitor signs of relapse via self-report, and to improve attendance rate for routine appointments and medication adherence [15–19]. One of these m-health technologies is the ‘Florence Telehealth’ system; a recent Kings Fund report (2018) summarised the adoption and spread of innovation in the NHS and concluded that ‘Florence’ had the potential to improve quality of life for patients with long-term conditions [20]. An adapted version of ‘Florence’ has now been developed for people with SMI; this intervention uses a newly developed simple interactive text messaging communication service.

This pilot study aimed to explore the feasibility and the potential clinical benefits of a SMI-specific mobile technology health management tool (‘Florence’) to enhance community care for people with SMI.

## Methods

### Study design and participants

This randomised-controlled pilot study used a mixed-method design to determine the feasibility of conducting a larger-scale randomised controlled trial (RCT) to evaluate the potential clinical benefits of using a mobile technology-assisted community care intervention (as compared with routine care for people with severe mental illness). Qualitative and quantitative data were collected at baseline and six months after the intervention.

There were two trial arms:

- Control group: received routine community mental health care under the Care Programme Approach (treatment as usual / TAU)
- Intervention group: received enhanced community care intervention that uses interactive SMS communication tools in addition to TAU.

### Intervention development, study setting, and recruitment procedure

The mental health specific mobile technology health management tool (‘Florence’) was developed through a co-production method (consultation group events) by a group of clinicians (Florence team members, consultant psychiatrist and psychologist, care coordinators) and patient experts by experience; this included the text messaging protocol, the wording for automated responses, agreements regarding the frequency for text message reminders for medication and appointments as well as putting together a user guide for patients.

The feasibility study was conducted within community mental health team settings in East London, involving two Community Mental Health / Recovery Teams and one Early Intervention in Psychosis team. Following a referral from clinicians, a research assistant arranged for a meeting to discuss study processes and the mobile technology intervention in detail, obtained written informed consent and conducted baseline assessments. Patients allocated to the intervention arm who did not own a mobile phone were provided with a mobile phone for the study.

#### Inclusion criteria

Eligible patients were those 18–65 years old who received mental health care from one of the community mental health teams (including Early Intervention in Psychosis teams) provided by East London NHS Foundation Trust.

Other criteria:

- Established diagnosis of Severe Mental Illness (Schizophrenia, Schizoaffective Disorder, Bipolar Disorder)
- Duration of illness ≥ one year
- Currently receiving care within the framework of the Care Programme Approach (be on CPA and have a care coordinator assigned to them or receive secondary mental care service in a depot clinic with regular reviews)
- Basic command of English

#### Exclusion criteria

- Lack of capacity (as assessed pre consent giving by patient’s clinicians)
- Organic psychosis
- Diagnosis of Learning Disability
- Currently an inpatient receiving acute care in hospital

### Intervention

The experimental intervention was an adapted version of the ‘Florence Simple Telehealth system’ (http://www.simple.uk.net), specifically designed for community care of patients with severe and/or enduring mental health problems. The ‘Florence’ text-messaging system (also referred to as ‘Flo’) was already available within the organisation (Community Health services in East London) and was used for supporting people with long-term physical conditions in the community. The manager of that service (experience in logistics and data handling) acted as a technical lead/advisor for the study. The intervention utilises the potential benefits of the ‘Florence’ technology for service user’s treatment adherence and therapeutic engagement. The research team created in co-production with service users an innovative mental health version for self-monitoring of relapse indicators, and for medication adherence reminders. The technology tools were designed to enable service users agreeing on three individually defined wellbeing indicators for their daily wellbeing scores, to monitor those in collaboration with health care professionals and ultimately foster communication between the service user and clinician outside routine appointments.

**Fig. 1:**
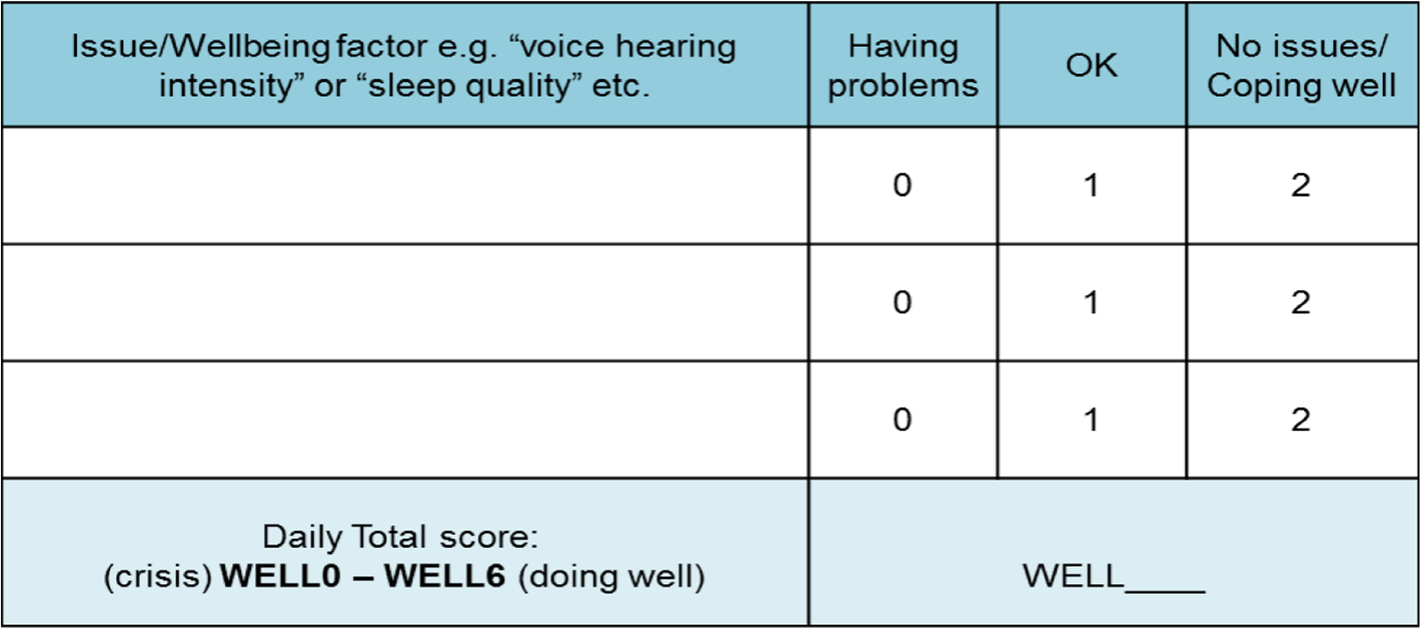
Wellbeing Matrix of individualised factors relating to wellbeing. Each item is rated on a 3 point Likert scale. The sum of scores for each factor determines the wellbeing score submitted to ‘Florence’ in the format *“WellX”*

A detailed description of the three intervention elements designed for the study project:

1. Medication/Wellbeing Reminders: ‘Florence’ sent service users four SMS text messages daily at individually specific times: two reminders for medication adherence or appointments and two asking service users to submit their wellbeing indicators
2. Wellbeing Indicator: Service users developed a personalised wellbeing indicator based on three individually defined issues/relapse signs (e.g. sleep, anxiety, voice-hearing intensity; see Fig. 1). Each item is rated as 0–2 (0 = having problems; 1 = minor problems; 2 = No issues/coping well), and the service user sends the sum-score of these three items (0–6, with ‘6’ indicating highest level of wellbeing) into the system on a daily basis. Depending on the scores received, automated response messages programmed into the ‘Florence’ system were provided. These included positive feedback (score 5–6; positive enforcement/encouragement), advice (score 3–4; how to self-support or access service support) or prompted service users to contact their care coordinator to discuss problems they experienced (score 0–2). See Table 1 for auto-response options for the wellbeing score.
3. Request Support Function: At any time, service users could use ‘Florence’ to send a message requesting support using a predefined list of help codes (see Table 1 for auto responses to help codes). In response, the care coordinator would contact service users to get more detailed information regarding the nature of the problem.

**Table 1:** ‘Florence’ Submission Auto-responses to Wellbeing Scores and Help Codes

**Wellbeing Score**	**Automatic response to wellbeing score submission**
5 or 6	Thank you. You’re doing really well. Take note of what you’re doing to help yourself. Flo
3 or 4	Thank you. Good to hear you’re coping well. Keep it up, Flo
2	Thank you. Sorry to hear things are difficult for you. You might want to look at your crisis plan to help yourself manage. Flo
0 or 1	Thank you. Sorry to hear you’re not feeling well. You might want to call the crisis line for advice on [local phone number given] or contact your care coordinator.
**Help Code**	**Automatic response to submitted request for help**
Mental health MH	Thanks for letting us know you’re having difficulties with your mental health. You can call the crisis line for advice if you need it: [local phone number given]
Physical health PH	Thanks for letting us know you’re having difficulties with your physical health. It may be best to book an appointment with your GP or attend A&E
Safety SF	Thanks for letting us know that you don’t feel safe. You can call the crisis line for advice on [local phone number given] or in an emergency call 999
Medication MED	Thanks for letting us know that you’re having problems with medication. You can call your GP or ask for an appointment with your psychiatrist to discuss this
Side effects SE	Thanks for letting us know that you’re having side effects. You can call your GP or ask for an appointment with your psychiatrist if you need to discuss this
Social contact SC	Thanks for letting us know that you’re having problems with social contact. Someone you trust might support you at this time if you talk it through with them
Finance FI	Thanks for letting us know that you’re having finance problems. You could try asking for advice from community links on [local phone number given]
HousingHO	Thanks for letting us know that you’re having housing problems. You could try asking for advice from [local area] Council on [local phone number]
Asylum (seeking) AS	Thanks for letting us know that you’re having Asylum problems. You could try calling RAMP for advice on [local phone number]
Employment EP	Thanks for letting us know that you’re having employment problems. You could try asking for advice from Workplace on [local phone number]

*Flo* Florence Intervention; *A&E* Accident and Emergency; *GP* General Practitioner; *RAMP* Refugee and Migrant Project; *Workplace* A local service offering employment advice

The intervention was introduced to the service user by the care coordinator and the research assistant, in addition, service users received a leaflet explaining the details of the intervention.

**Fig. 2:**
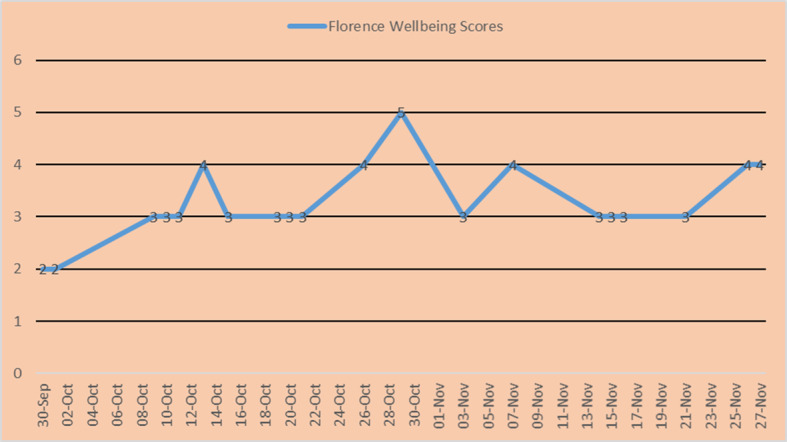
Example of an auto-plot of self-rated wellbeing scores over time

The electronic messaging system enabled online monitoring of the wellbeing scores on a dashboard (see example in Fig. 2). If a service user gave a relapse indicator score of 0–1 (i.e. indicating some relapse signs) the care coordinator was requested to arrange for a telephone/Skype session or face-to-face contact with the person. The service user would be contacted within 24 h (weekday) or on the first day of the week if the message was submitted on weekend days. Service users were clearly advised that the ‘Florence’ system would not replace the usual acute and crisis care arrangements.

The control condition was Treatment as Usual (TAU), i.e. standard community mental health care. TAU involved routine follow up with one monthly face-to-face contact by any mental health care practitioner (usually the care coordinator) and 3–6 monthly medical reviews with a psychiatrist.

**Outcome measures** (all at baseline and six months after the intervention was completed).

We collected data for baseline characteristics from electronic patient record systems. Data included sociodemographic information, diagnoses and ICD codes, care cluster codes, medication prescribed, number of previous hospitalisations, date of the last hospitalisation, duration of illness, and history of relapse due to non-compliance.

The primary outcome measure was the DIALOG scale (PROM: patient-reported outcome measure) [21] at baseline and after six months. DIALOG captures patient satisfaction scores on domains representing subjective quality of life measures (SQOL) components of the DIALOG+ intervention [22]. There are eight SQOL domains, these are mental health, physical health, job situation, accommodation, friendships, leisure activities, partner/family, and personal safety. These are rated by the patient on a Likert scale ranging from 1 (couldn’t be worse) to 7 (couldn’t be better). Using the same Likert scale within DIALOG an additional three items measuring treatment satisfaction were scored: medication, practical help received and meetings with professionals (PREM: Patient Reported Experience Measure).

Secondary outcome measures were measured at baseline and after six months:

1. Intervention adherence: Recorded by measuring SMS response rates on the ‘Florence’ system. This involved recording daily wellbeing scores as well as the patient’s text inputs into the system.
2. Treatment adherence: Recorded by assessing compliance with medication using the Medication Adherence Rate Scale (MARS) [23, 24]; the scale consists of 10 items and patients rate the answer as “Yes” or “No”; The total score ranges from 0 to 10. with a higher score indicating better adherence.
3. Patient satisfaction with treatment: Recorded using the Client’s Assessment of Treatment Scale (CAT), [25]; the scale assesses patients’ subjective satisfaction and perceptions of their treatment using 7 items (e.g., “do you believe you are receiving the right treatment/care for you here?”). Each item is rated on a visual analogue scale with 11 points with scale endpoints that ranged from 0 (‘not at all’) to 10 (‘yes entirely’).
4. Factors contributing to effective self-management skills were measured using two validated scales:

- General Self Efficacy Scale [26]; a self-report measure of self-efficacy with 10 items, total score is calculated as the sum of the items and ranges between 10 and 40, with a higher score indicating more self-efficacy. Measures were reported as mean scores (range 1–4).
- Mental Health Confidence Scale [27]; a 16-item scale for patients to rate their degree of confidence on a 6-point Likert scale from “1 (very Non confident) to 6 (Very confident). The total score ranges from 16 (very low confidence) to 96 (high level of confidence).

### Randomisation

Once written informed consent had been obtained, eligible patients were randomly assigned to TAU or the intervention in addition to TAU. Random assignment of group allocation was conducted using concealed consecutive numbers (computer-generated), provided by an independent academic unit staff member not involved with the trial. Both conditions were delivered in the community. Masking was not possible due to the nature of the experimental intervention.

### Sample size

Given the exploratory nature of the pilot study, we did not conduct a sample size calculation but followed the principles established in the wider literature (flat rules of thumb for overall pilot trial sample size of a two armed trial); we decided to aim for a sample of > 50 participants (> 25 per arm) as recommended for standard effect sizes that are small to medium [28].

### Assessment of the feasibility of the RCT

The following criteria were set before the study started to indicate that both the study and intervention were feasible for a full RCT:

1. Recruitment of target sample size within the given recruitment period of consecutive 12 months, i.e. 5 participants per month
2. Engagement of 80% of participants allocated to the ‘Florence’ intervention.
3. Retention of 60% of participants for the ‘Florence’ intervention.
4. Follow-up data available from 70% of participants on the primary outcome at six months

### Ethics approval and consent to participate

Ethics approval was obtained from the London-Camden & Kings Cross Research Ethics Committee (Health Research Authority) Reference: 16/LO/1117, Protocol number: R-403-668, IRAS project ID: 205395. All methods were performed in accordance with guidelines and regulations and all participants provided written informed consent to participate.

### Statistical analysis

Descriptive statistics were completed for all of the outcome data and presented separately for treatment arms at baseline and follow-up. We conducted t-tests to compare baseline sociodemographic and clinical baseline characteristics of the two groups. All analyses were conducted using IBM SPSS statistics 25 for Windows. The feasibility trial aimed to evaluate the acceptability and viability of the health technology intervention in this patient group and it was not powered to detect significant differences. To evaluate the relative effects of the two treatment conditions, univariate analyses of covariance (ANCOVAs) for each of the outcome measures (DIALOG PROM and PREM, General Self Efficacy sale, Mental Health Self Efficacy Scale and Medication Adherence Scale) were performed, using the pre-treatment scores as covariates. Pearson’s correlation was used to examine correlations among wellbeing scores and outcome measures.

### Qualitative study

The qualitative part of the study was designed to understand how patients and staff experienced using the intervention and being part of the pilot trial. Information regarding experiences using the intervention and acceptability of the ‘Florence’ system was obtained through individual semi-structured interviews at follow-up with patients (*N* = 31 completed). Interviews were also conducted at baseline with patients in both arms of the trial (25 in the control and 31 in the intervention arm) to determine how patients experienced care as usual.

The semi-structured interviews took place in the community at the team base or patients’ home (according to their preference); they were conducted using a topic guide and were recorded (transcribed verbatim) by the two study research assistants. The participants had no prior relationship with the researchers. The researchers were psychologists by professional background and trained in conducting interviews, and written consent was obtained before the interview began. Additional data were collected from staff (*N* = 21) via email circulation of a questionnaire which asked open questions about their experience of ‘Florence’.

Analysis of the qualitative data (by DM and SB) took place after the quantitative analysis was completed. Interview recordings were subjected to thematic analysis [29], coded initially (by DM) using NVivo version 12 qualitative data analysis software. All data were thematically coded by a second experienced qualitative interviewer (SB), and results were discussed by both to conclude the final thematic analysis.

## Results

### Participant flow and data

**Fig. 3:**
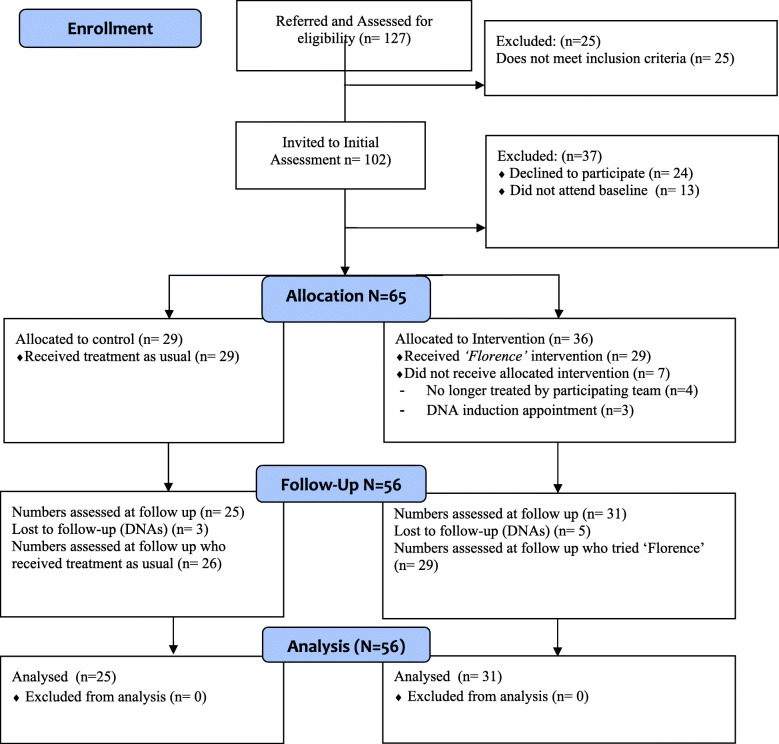
Consort Flow Diagram

A total of 127 referrals were made for enrolment into the trial, 102 were eligible for inclusion and invited to a further assessment. A portion of participants either did not attend the baseline assessment (*N* = 13) or declined to participate in the trial (*N* = 24) leaving 65 participants eligible for allocation, see the Consort study flow diagram (Fig. 3). Patients were recruited from Community Mental Health Teams/Recovery teams (*N* = 42) and Early Intervention in Psychosis teams (N-23). Eligible participants were recruited over 11 months (August 2016 to June 2017). The observational period for both arms was six months from baseline assessment (last follow-up assessments conducted in January 2018). No losses or exclusions at randomisation were recorded. There was only one protocol deviation, different to the first protocol version; we also decided to include patients from an enhanced primary care service associated with the Community Mental Health teams, provided they fulfilled all inclusion criteria.

### Assessment of the feasibility of the RCT

The study met the four predefined criteria to assess the feasibility of the RCT. The number of people recruited exceeded the target sample size of five participants a month; the rate of recruitment was 5.9 people per month. For the participants allocated to the Florence intervention, 89% of the participants engaged with Florence (80% engagement threshold set) and 56 participants (86%) were retained in the study (60% retention threshold set), and for these follow-up data were obtained (70% follow-up threshold set).

### Baseline participant characteristics

The total participant group consisted of 34 female and 31 males with a mean age of 35.2 years. The mean age of leaving education was 19.9 years. The ethnicity of the group was broadly in keeping with the expected population percentages for the locality in East London: 19.4% white Caucasian, 17.9% Black African, 16.4% Bangladeshi, 13.4% Pakistani, 10.4% Black Caribbean, 7.5% Indian, and 6% Black other.

Nearly half of participants (46%) were diagnosed with Schizophrenia (*N* = 31; ICD10 F20), predominantly paranoid schizophrenia subtype. Other diagnoses included: 21% bipolar affective disorder (*N* = 14; F31), 15% psychotic disorder unspecified (*N* = 10, F23), 12% schizoaffective disorder (*N* = 8; F25) and 3% delusional disorder (*N* = 2; F22).

Sociodemographic and clinical baseline characteristics of the two groups are included in Table 2. Both groups were well matched in their baseline characteristics, t-tests revealed no significant differences between groups.

**Table 2:** Baseline characteristics and clinical data of participants who entered the Flo trial

	‘Florence’ + TAU	TAU	t-test
	**(*N*** **= 37)**	**(*N*** **= 28)**	**(Df = 62/63)**
**Gender female, n (%)**	19 (51)	15 (54)	–
**Age, mean years (sd)**	36.2 (11.4)	33.9 (10.9)	n.s.
**Duration of illness, mean years (sd)**	16.3 (11.3)	12.1 (9.2)	n.s.
**Age leaving education mean age (sd)**	24.4 (18.7)	28.1 (25.3)	n.s.
**Number of previous hospitalisations, mean number (sd)**	2.6 (2.5)	2.3 (2.6)	n.s

*n.s.* not significant; *sd* Standard deviation; *TAU* Treatment as usual

### Utilisation of the intervention elements

None of the participants make use of the Request Support Function at any moment in time, suggesting that this element of the intervention was not sufficiently explained and / or introduced. All patients on medication received daily two reminders for their oral maintenance medication.

The Wellbeing Score element of the intervention was utilised by 27 of the 29 participants who received the intervention; *N* = 20 patients used the wellbeing monitoring consistently every month of the six-month intervention period. The range of wellbeing scores sent into the Florence monitoring system per month was 5–27 scores, 20 participants sent their score at least on 50% of days (minimum of 15 per month), the mean number of wellbeing scores per month was *N* = 16.6. Eleven participants continued to use the wellbeing scoring feature for another 2–6 months after they completed the trial intervention (total number of wellbeing scores for these participants ranged from 57 to 327).

### Quantitative outcome data analysis

The data analysis did not identify significant changes over time in favour of the ‘Florence’ intervention arm in any outcome; clinical outcomes are summarised in Table 3. Most patients had on average relatively high baseline scores for primary and secondary outcome domains (representing relative higher satisfaction with subjective Quality of Life domains, as well as a higher level of self-efficacy, self-confidence and medication adherence).

**Table 3:** Clinical outcome measures (ANCOVAs, adjusted for baseline score)

	‘Florence & TAU’			TAU			Difference (95% CI)
	N	Mean	s.d.	N	Mean	s.d.	
**DIALOG PROM mean score 8 items**							
- at baseline	30	4.6	0.8	24	4.3	1.4	
- post-treatment	30	4.8	1.1	24	4.6	1.2	−0.27 (−0.51 to −0.02) *
**DIALOG PREM mean score 3 items**							
- at baseline	31	5.6	0.9	25	5.1	1.4	
- post-treatment	31	5.7	1.0	25	5.3	1.0	−0.14 (−0.49 to 0.20) n.s.
**General Self Efficacy Scale (GSE)**							
- at baseline	30	2.8	0.7	23	2.7	0.8	
- post-treatment	30	2.7	0.7	23	2.8	0.6	0.16 (−0.17 to 0.20) n.s.
**Mental Health Confidence Scale**							
- at baseline	29	65.4	15.4	24	61.8	19.5	
- post treatment	29	62.4	17.2	24	66.5	15.5	−0.85 (−4.97 to 3.23) n.s.
**Medication Adherence Scale (MARS)**							
- at baseline	31	7.7	2.1	25	7.6	2.7	
- post-treatment	31	7.6	2.3	25	7.7	2.1	−0.14 (−0.79 to 0.50) n.s.

DIALOG PROM: Patient Reported Outcome Measures, mean across 8 domains (mental health, physical health, job situation, accommodation, leisure activities, relationships, friendships, personal safety); DIALOG PREM: Patient Reported Experience Measures, mean across 3 domains (satisfaction with: medication, practical help, meetings); *n.s* not significant; *TAU* Treatment as usual; * The mean difference is significant at the .05 level.

*Each Scale is positively ranked where higher scores indicate higher satisfaction/efficacy/confidence/adherence

The statistical analysis revealed that patients mean wellbeing scores over time were highly positively correlated with all main outcome measures (sum-scores at follow-up; Pearson’s correlation coefficients of 0.58–0.68).

Results from the Client’s Assessment of Treatment (CAT) scale indicated high level of satisfaction with treatment in general, understanding of health professionals, medication (mean scores in both groups of 7.0–8.2; s.d. 1.6–3.2; on scale between 0 and 10).

### Qualitative interview data (findings from thematic analysis)

The findings presented here include service user and staff responses from the interviews. We present data that gives indication of how Florence may have influenced care in addition to treatment as usual. Overall, the findings from the qualitative interviews are organised around three main themes 1) the impact of the ‘Florence’ intervention on routine community mental health care 2) the acceptability of the Florence intervention amongst servicer users and community mental health professionals 3) recommendations for future development of the Florence intervention. The thematic analysis is presented below, further examples of illustrative quotes for each theme can be found in Table 4.

**Table 4:** Themes, sub-themes and illustrative quotes from thematic analysis

Theme 1: The impact of the ‘Florence’ intervention in community mental health care		
Subtheme	Quotes from Service Users	Quotes from clinicians
1.1: ‘Florence’ facilitating service user-clinician communication	“Florence worked well, like someone checking in, and getting the text message back giving advice or saying you’re doing well was helpful. I was looking forward to the message, like a friend.” [Flo, SU, 009]	“Perfect intervention to communicate their well-being or otherwise to their CCO regularly”. [Flo, CC, 007].
1.2: The value of ‘Florence’ medication adherence reminders	“I just like it cause it’s so helpful. Particularly if you’re having a bad day as I would forget meds, mainly the evening ones when feeling low so having the reminders was helpful.” [Flo, SU, 025]. “Brilliant. A few times where I was busy, it would remind me to take my medication” [Flo, SU, 025] “It changed a lot because before, a few times I used to forget to take the medications but now it prompts me to take the medications at the right time so there’s no complications.” [Flo, SU, 050]	“Would be really good for clients when they are coming out of hospital for at least a week or maybe a month to help compliance with medication. Especially for people who have regular admissions after non-compliance. I also think it would be helpful for GP surgeries when medications are first prescribed to help for compliance and monitoring”. [Flo, CC, 063]
1.3: Florence supporting service users and clinicians to focus on feelings	“It’s very nice that the system also asks how I’m doing every day on a scale of 0–6 so it makes me think about how I’m doing all the time. It’s like mindfulness, it helps me to be aware of how I’m doing and it like helps me to be mindful” [Flo, SU, 050]	“… she’s been using it and found it very helpful to track her mood. I’ve been checking in regularly and it’s helped me to be aware of how she’s doing”. [Flo, CC, 021]
Theme 2: The acceptability of the Florence intervention amongst servicer users and community mental health professionals		
Subtheme	Quotes from Service Users	Quotes from clinicians
2.1: Satisfaction with Florence	“No, I think it was all good, overall, I don’t have any complaints. With the ‘Florence’ system I don’t think there’s anything that could be improved, I think it’s perfect.” [Flo, SU, 062] “Makes you feel less isolated and that you’re being monitored when not feeling well […] I feel well supported by the team and [NHS] Trust but not my family. The Trust could use FLO to help with support and guidance which would be useful. “[Flo, SU, 009].	“Positive she was happy” [Flo, CC, 47]
2.2: Usability of Florence	“The questions were good, straight forward, you just reply with the number that you’re feeling. It was very easy to use” [Flo, SU, 050] “I liked the graph which I think was helpful for Y and she showed it to me sometimes too. The replies of the messages was always positive and I appreciated them most days, they made me smile.” [Flo, SU, 021] “… 10 people with the same diagnosis as me will have 10 different ways of experiencing the illness. So I like that it’s specific to me, it makes me feel more in control. I like that you can change it to your specific needs.” [Flo, SU, 063]	“Flagging up when a service user is not doing well is helpful” [Flo, CC, 025]
Theme 3: Recommendations for the development of the Florence intervention		
Subtheme	Quotes from Service Users	Quotes from clinicians
3.1: Personalisation of Florence	“… 10 people with the same diagnosis as me will have 10 different ways of experiencing the illness. So I like that it’s specific to me, it makes me feel more in control. I like that you can change it to your specific needs.” [Flo, SU, 063] “It would be good to be able to adjust the frequency so by continuing on the same score until I submit a different score.” [Flo, SU, 031]]	‘Individualising of text messages for each patient, appointment reminders’ [TAU, CC 66]
3.2. Practical considerations implementing ‘Florence’		“A prompt for clinicians to discuss the intervention with patients would be helpful.” [Flo, Con, 007]

*SU* Service user; *CC* Care coordinator; *Flo* ‘Florence’ intervention; *TAU* Treatment as usual

#### Theme 1: the impact of the ‘Florence’ intervention in community mental health care

Service users in the intervention arm of the trial highlighted several ways that the clinical care was impacted by the use of the ‘Florence’ intervention, these included: 1) ‘Florence’ facilitating communication with clinicians 2) valuing medication reminders, and 3) helping people to focus on feelings.

##### Florence facilitating service user-clinician communication

Most participants felt that the structure of care they received did not change as a result of using ‘Florence’, however, a few service users reported an increase in the number of meetings with their care coordinator and more responsive care (e.g. a low wellbeing score led to a phone call from a clinician). Some participants spoke about conversations that had been initiated with their care-coordinator about using ‘Flo/rence’ and agreeing on collaborative working. Frequent contact with the clinical team or knowledge that they could contact them if they needed was important for several participants. This was recognised as providing comfort and demonstrating that there was consistent support available from the healthcare team, emphasis was placed by some on the specific advice provided by clinicians.

*“Probably constant communication, a good care coordinator who understands me for when I feel like I’m going off the rails. I can email them, and they respond well. So, email and understanding from care coordinator.” [Flo, SU, 021].*

Service users also spoke about valuing the quality of the therapeutic relationship with their healthcare team; *‘they’re a really good team’*, the demonstration of support and understanding was recognised as integral to the interaction.

##### The value of ‘Florence’ medication adherence reminders

A large proportion of participants who had trialled the ‘Florence’ intervention had an overwhelmingly positive experience of receiving medication reminders. A few participants indicated that the ‘Florence’ system had helped them to get into the routine of taking medication and that thereafter they no longer needed the medication reminders. One clinician commented that a participant had been able to take more responsibility for their medication which had enabled them to function more independently from family members and develop mutually supportive relationships:

*“[Name] has been able to individuate from [their] mother, by taking more responsibility for [their] own care, and this in turn has led to their relationship being more some symbiotic; mutually supportive.” [Flo, CC, 009].*

Another clinician suggested that ‘Florence’ might be a useful tool for promoting self-management skills in periods of vulnerability and reduced function, such as on discharge from inpatient care and may not be necessary as a long-term intervention.

##### Florence supporting service users and clinicians to focus on feelings

Most participants described an initial positive experience of reflecting on their daily functioning by giving a wellbeing score, some found this more cumbersome over time, leading to reduced frequency or discontinued responses. Many participants felt that it was helpful to be asked how they are on a daily basis and be prompted to reflect on their wellbeing. The ability to monitor progress using graphs alongside receiving positive messages on a regular basis was valued, encouraging empowerment and addressing power imbalances between service users and clinicians:

*“The message, well 3, “thank you, I’m glad you’re coping well is very nice”. It’s tailored to your needs.” [Flo, SU, 028].*

A clinician commented on how it’s helped a service user to keep track of mood and increased her awareness of their well-being. Another clinician commented on the impact of the well-being aspect of ‘Florence’ on recovery:

*“It also helped them to think about how they were doing from day-to-day in themselves which in turn helped them further develop their reflective capacity and therefore recovery.” [Flo, CC, 009].*

Some participants found the scoring a little challenging and acknowledged that sometimes they censored their responses and at other times avoided contact with their care coordinator. Some felt cautious about being monitored *‘they never used to check up on me before,”* whilst others acknowledged this could be a useful gateway to accessing care and averting crises.

#### Theme 2: the acceptability of the Florence intervention amongst servicer users and community mental health professionals

##### Satisfaction with Florence

Service users and staff were broadly satisfied with the utility of the text messaging tool, 27 out of 42 staff (64%) thought that ‘Florence’ would be a useful service for their clients and generally, clinicians thought that some service users would be open to using this digital technology:

*“yes, people expressed they would like to be asked about their mood and perhaps use the ‘Florence’ similarly to a mood diary. It seems useful for people who are actively looking to engage with their mental health.” [TAU, CC, 066].*

Service user participants were asked if they would continue using the system, 16 people (64%) said they would continue using the ‘Florence’ system. Several people expressed that that system was ‘excellent’, and one participant thought it should be ‘scaled up’:

*“They should use this on a larger scale, more frequency of texts.” [Flo, SU, 009].*

Some participants expressed retrospectively how they were pleasantly surprised by the impact of this intervention and that they were still using it. Some were surprised to see a quick response from their clinician when a low score was recorded:

*“The first time I got a call from [name of care-coordinator] when I scored really low and she called me I was quite surprised because I didn’t know that they got the feedback.” [Flo, SU, 046].*

Similar to service user perspectives, one clinician also discussed the helpfulness of the alert when well-being scores were very low, supporting access to care and preventing crisis:

*“Flagging up when a service user is not doing well is helpful in Flo” [CC, 025].*

Other clinicians highlighted it could be useful in helping service users to develop self-management skills, that medication reminders were well received, that it was simple to use, and that it could act as a conduit between the service user and the care coordinator.

##### Usability of Florence

Some service users reported that they were hesitant about using the ‘Florence’ system at first and were uncertain about its utility, however, acknowledged that they adjusted to the system and felt it was *‘fairly straight forward’* with easy to read and clear questions. One person said that they did not use all the functions of ‘Florence’ such as the help codes and questioned their understanding of using the system. Some of the respondents who wanted to continue using the system offered ways they would like to tailor the use whilst a few participants spoke about technical challenges experienced (e.g. scores not being received by the care-coordinator).

#### Theme 3: recommendations for the development of the Florence intervention

Service user participants made recommendations about how the ‘Florence’ system could be adapted for future use in the services.

##### Personalisation of Florence

Personalisation of the system based on the service user’s individual need and their daily routines was important to many of the participants. Some people felt that the frequency and timing of completing wellbeing scores could be adapted to be less or more frequent depending on individual need. The time that they received wellbeing score reminders was not always suitable for many respondents:

*‘10 in the morning it’s too early and some people’s days haven’t started’*.

Another participant suggested alternative formats for the wellbeing scores such as using an emoji, whilst another wanted to reply with more detail and put how they were feeling in words rather than using a score:

*“It would be good to be able to text back something else, like words. I know there were a list of words that I could have text back, but I really want to text back what I’m feeling. [ …*] *Every number I know corresponds to how I was feeling. 6 was I’m on top of the world and 0 meant I’m going to go and jump off a bridge, but what does 3 mean …*? *” [Flo, SU, 063].*

Some expressed that they would like more flexibility or control of the functions, such as be able to submit well-being scores after 10.30 pm or the ability to tailor the timing of reminders.

##### Practical considerations of implementing ‘Florence’

Some members of staff provided recommendations for the usefulness of ‘Florence’ whilst others expressed specific use for ‘Florence’, such as when supporting service users to make transitions, or for use in an outreach format rather than offered as part of routine care. One staff member expressed concerns about the automated nature of the system:

*“Service users to have more responsibility and power on sharing their information with clinicians, therefore no automatic response, but a choice of whether to share the last weeks or days score with care coordinator. This may increase service user autonomy.” [Flo, CC 25].*

One clinician felt that more guidance and a prompt to remind them to discuss ‘Florence’ with their service users should be considered. Whilst others described contextual barriers they experienced whilst implementing Florence, such as the perceived increase in team workload alongside the consideration of other digital technology being trialled within the care offer.

## Discussion

To our knowledge, this is the first randomised-controlled trial to investigate the clinical utilities of a ‘Simple-Telehealth’ (Florence) text-messaging technology to assist the mental health care of patients with severe and enduring mental illness. Preliminary evidence from small cohort and case studies suggests that “Florence is a low-cost, low-risk innovation with a strong track record” [30]; studies reported benefits on self-management, recovery outcomes and treatment adherence, predominantly in the management of physical long-term conditions such as hypertension and diabetes [31], but also more recently for patients with mental health problems following psychotherapy [32]. Other studies provided preliminary evidence for the effectiveness of more complex text-messaging interventions that incorporated Cognitive Behaviour Therapy techniques to target medication adherence and psychotic symptoms [33] or used an ‘Early Warning Signs Questionnaire’ for relapse prevention [34].

### Study findings

The overall aim of this randomised controlled pilot study was to explore the feasibility and the potential clinical benefits of the ‘Florence’ mobile health management tool for people with SMI.

Trial findings demonstrate that it is feasible to implement the intervention within this patient cohort and that the study design can be delivered. All four pre-set criteria to assess feasibility were met; attrition rates were acceptable and within normal limits, and the study demonstrated subjective benefits (clinical significance) by facilitating recovery-oriented community mental health care for patients with severe and enduring mental illness. No harmful effects were observed as a result of the trial. Those who were in the TAU arm showed enthusiasm to use the intervention as well.

Qualitative data collected from staff also showed that there was a lot of interest in supporting the implementation of the project by clinicians; however, some staff did raise resource concerns. An enabler of the pilot trial was the support received by sponsors in promoting the research within the clinical teams where the study took place. Support provided by the Telehealth team was pivotal, escalating messages to the care coordinators as required and also concerning teams’ decision making to continue using ‘Florence’ beyond the research project.

One feature of the intervention was not utilised as per protocol; this was the interactive messaging option concerning unmet needs (help codes). Follow-up interviews revealed that a significant contextual challenge impacted upon the delivery of this aspect of the intervention: following a service redesign, local teams had undergone relocation and merger from four separate teams to two teams only three months before the recruitment phase. Although it is difficult to quantify the impact that this had on engagement and recruitment, it is likely to have influenced staff morale and motivation to improve client-practitioner communications, as there was concern that this may increase the workload further. A future trial needs to strengthen the health professional involvement for the delivery of this element (e.g. revised set-up procedures and training package).

Results for the primary subjective quality of life outcome measure (DIALOG) significantly increased from baseline to follow-up, but the study did not detect significant differences between the experimental and TAU groups for the primary or secondary recovery outcome measures over time; this has been associated with the fact that baseline scores for all outcomes were relatively high and therefore indicating that a more stable cohort with lower levels of dissatisfaction was recruited to participate. This ceiling effect in the participant’s subjective quality of life, self-efficacy/−confidence and treatment adherence levels left little room for further improvement. The participants however had been in need for service support for many years (average duration of illness 16 years) and indicated some significant benefits using the telehealth intervention, particularly self-monitoring of wellbeing and medication reminders. It is possible that more noticeable changes in the outcome measures may be found in participants in different stages of recovery; patients who have just accessed mental health services in crisis such as those experiencing first-episode psychosis or a psychotic relapse/breakdown or profound chronic symptoms are likely to have lower baseline scores.

Patients valued the (twice daily) medication reminders, reporting that this would be particularly helpful if they had a difficult day, thereby suggesting that the reasons for non-adherence were predominantly unintentional and not a result of negative attitudes towards maintenance therapy. The medication reminders helped to develop a daily routine for some participants. Similarly, a recent systematic review found that patients with unintentional non-adherence may benefit from automated text messages or app reminders [35].

Participants reported that the tool could enhance the therapeutic relationship and maintain continuity in care. This is a topic that requires further exploration in research, given the ongoing debate [36] about the impact of telemedicine on the therapeutic alliance within different patient populations and taking into account the varying therapeutic objectives.

The fact that the wellbeing scores were highly correlated with recovery care outcome measures suggests that a relatively simple and individually defined wellbeing score is highly predictive of a range of outcomes relevant for the provision of recovery care services for severely mentally ill patients and could be usefully easily implemented into routine care. Wykes & Brown [37] conducted a risk-benefit analysis of e-health tools and interventions, emphasising the importance of skilled responses from clinicians to self-monitoring data. The approach chosen for the self-monitoring of wellbeing in this study with a self-defined score based upon three most relevant relapse indicators has significant advantages; the resulting (positively coded) wellbeing scores reliably identified those patients who require more immediate support (scores of 0–1). This allows the allocation of limited resources in a more clinically informed and cost-effective manner. Some patients found the request for a wellbeing score helpful for making them more aware of their wellbeing and for identifying relapse. The clinicians also reported that ‘Florence’ could help patients to develop reflective capacity. This is different to tools evaluated in other studies, where an automated telehealth device (e.g. ‘Health Buddy’ or ‘MONARCA’ system) operates on the basis of a predefined content library to determine symptoms for self-monitoring [38, 39].

The main recommendation from patients was that the intervention could be customised to meet individual preferences, some study participants reported that they became either quickly fatigued by the frequency of messages or felt that they were too intrusive. Similar reports have been raised in the literature [35]. A desire to have more autonomy over the use of the ‘Florence’ tools was also emphasised by one of the staff participants. Empowerment is one of the key aspects of recovery-focused care and therefore, should feature in technology-assisted self-management tools aimed at supporting recovery and enable patients to exercise more choice and control [36]. The option to customise the intervention would ensure that the intervention was more personalised and maybe more easily implemented using text-messaging or an app. Equally, Basit et al. [35; p. 35] recommended to explore patients’ preferences for the type of technology tool used for medication reminders and to “focus on patients with known adherence problems and stratify them based on the reason for non-adherence (e.g., intentional and unintentional)”.

Staff raised concerns about the workload implications to support the implementation of ‘Florence’. Similar concerns have been raised in other studies using telemedicine, and they are highly dependent on the level of intensity/interaction of the intervention [35–39]. ‘Florence’ is an intervention which mainly uses automated text messaging but also includes a monitoring and follow-up element via telephone if relapse indicators are evident. Telemedicine interventions have been previously categorised into levels of intensity. Interventions solely using automated text messaging have been classified as low-intensity interventions; however, when automated text messaging is paired with tele-monitoring and response to alerts the intervention is considered a medium intensity intervention [35].

A potential barrier for the adoption of telehealth interventions in this population is the ‘digital divide’, i.e. disadvantaged populations who do not have access and use of digital media; previous studies reported that access to mobile technology is significantly lower than within the general population [40]. The two challenges with implementing telehealth solutions in this population are skills and access [37]. The provision of a mobile phone for the participants in this study was a solution aimed at overcoming the problem of physical access, the potential further barrier around skills acquisition may require more attention for people to engage fully with the phone functions. ‘Florence’ Simple-Telehealth tools however appeared to have been easily adopted by the majority of participants in this study, patients reported that the intervention was easy to use.

For the purpose of this study a simple text messaging technology was chosen with a view to maximise chances for patient inclusion. A text-message-only system offers the lowest threshold for access to the service but limits the sophistication and personalisation. In future, an app-based version of the intervention could be tested with enhanced interactive features, using a stepped-care approach as suggested by Basit et al. [35].

### Strengths and limitations

A strength of the study was that there was a sizeable qualitative component to this pilot study, with interviews with patients and clinicians which enable experiences and suggestions for future recommendations of using Florence to be captured. In addition, this adapted version of ‘Florence’ was developed co-produced with patients to ensure that the automated responses were relevant to people who received care. Pre-set criteria were also used to assess the feasibility of the study; this was complemented by the qualitative data.

A relevant limitation of the study was that people involved in the trial had been in contact with mental health services for several years and presented in a more stable stage of recovery at baseline, resulting in a ceiling effect on the outcome measures. Our data, therefore, does not indicate how someone relatively new to services and patients with severe clinical symptoms and or problems with health/social care engagement would experience this intervention and how this would support their recovery. Equally, the intervention might be best suited for patients who receive crisis care and subsequent periods of rehabilitation in the community.

## Conclusion

In conclusion, recruitment to the trial was feasible, and a full trial could go ahead. In follow-up trials the inclusion criteria should be amended and include patients with higher level of need (lower baseline scores for PROMs, Recovery indices and Medication Adherence) and clinicians should receive a tailor made, dedicated and more in-depth induction to the intervention tools and receive assistance in managing the tools. Patients indicated that they would want to further personalise the intervention and a full trial is required to examine potential longer-term benefits as well as adverse effects associated with the intervention in a larger sample size. Clinically, the main utility of this intervention is the user-defined measure of wellbeing to support patient’s capabilities for self-monitoring and to foster their empowerment, secondly the medication reminders as an aiding tool for treatment adherence and potentially the opportunity for meaningful feedback about experienced care needs.

## Data Availability

The datasets used and/or analysed during the current study are available from the corresponding author on reasonable request.

## Abbreviations

SMS: Short Messaging Service
TAU: Treatment as Usual
PROM: Patient Reported Outcome Measure
SMI: Severe Mental Illness
NHS: National Health Service
RCT: Randomised Controlled Trial
ICD: International Classification Disease
SQOL: Subjective Quality Of Life
PREM: Patient Reported Experience Measure
MARS: Medication Adherence Rating Scale
CAT: Clients Assessment of Treatment
SU: Service User
CC: Care Coordinator
FLO: Florence
SPSS: Statistical Package for the Social Sciences
ANCOVA: Analyses Of Covariance
